# Total intravenous anesthesia with Ketofol in rabbits: a comparison of the effects of constant rate infusion of midazolam, fentanyl or dexmedetomidine

**DOI:** 10.1186/s12917-024-04112-w

**Published:** 2024-06-08

**Authors:** Arghavan Mofidi, Nasser Vesal

**Affiliations:** Department of Veterinary Clinical Sciences, School of Veterinary Medicine, Shiraz University, Shiraz, 71441-69155 Iran

**Keywords:** Dexmedetomidine, Fentanyl, Ketamine, Midazolam, Propofol, TIVA, Rabbit

## Abstract

**Background:**

When inhalant anesthetic equipment is not available or during upper airway surgery, intravenous infusion of one or more drugs are commonly used to induce and/or maintain general anesthesia. Total intravenous anesthesia (TIVA) does not require endotracheal intubation, which may be more difficult to achieve in rabbits. A range of different injectable drug combinations have been used as continuous infusion rate in animals. Recently, a combination of ketamine and propofol (ketofol) has been used for TIVA in both human patients and animals. The purpose of this prospective, blinded, randomized, crossover study was to evaluate anesthetic and cardiopulmonary effects of ketofol total intravenous anesthesia (TIVA) in combination with constant rate infusion (CRI) of midazolam, fentanyl or dexmedetomidine in eight New Zealand White rabbits. Following IV induction with ketofol and endotracheal intubation, anesthesia was maintained with ketofol infusion in combination with CRIs of midazolam (loading dose [LD]: 0.3 mg/kg; CRI: 0.3 mg/kg/hr; KPM), fentanyl (LD: 6 µg/kg; CRI: 6 µg/kg/hr; KPF) or dexmedetomidine (LD: 3 µg/kg; CRI: 3 µg/kg/hr; KPD). Rabbits in the control treatment (KPS) were administered the same volume of saline for LD and CRI. Ketofol infusion rate (initially 0.6 mg kg^− 1^ minute^− 1^ [0.3 mg kg^− 1^ minute^− 1^ of each drug]) was adjusted to suppress the pedal withdrawal reflex. Ketofol dose and physiologic variables were recorded every 5 min.

**Results:**

Ketofol induction doses were 14.9 ± 1.8 (KPM), 15.0 ± 1.9 (KPF), 15.5 ± 2.4 (KPD) and 14.7 ± 3.4 (KPS) mg kg^− 1^ and did not differ among treatments (*p* > 0.05). Ketofol infusion rate decreased significantly in rabbits in treatments KPM and KPD as compared with saline. Ketofol maintenance dose in rabbits in treatments KPM (1.0 ± 0.1 mg/kg/min) and KPD (1.0 ± 0.1 mg/kg/min) was significantly lower as compared to KPS (1.3 ± 0.1 mg/kg/min) treatment (*p* < 0.05). Ketofol maintenance dose did not differ significantly between treatments KPF (1.1 ± 0.3 mg/kg/min) and KPS (1.3 ± 0.1 mg/kg/min). Cardiovascular variables remained at clinically acceptable values but ketofol infusion in combination with fentanyl CRI was associated with severe respiratory depression.

**Conclusions:**

At the studied doses, CRIs of midazolam and dexmedetomidine, but not fentanyl, produced ketofol-sparing effect in rabbits. Mechanical ventilation should be considered during ketofol anesthesia, particularly when fentanyl CRI is used.

## Introduction

General anesthesia in rabbits is usually induced with injectable agents and maintained with inhalation anesthetics in oxygen delivered by mask or endotracheal tube. Although inhalation anesthesia is typically the first choice when a long duration of anesthesia is required, there are some drawbacks including the danger of chronic exposure of operating room personnel to low concentrations of volatile anesthetic agents and the need for a suitable anesthetic machine and endotracheal intubation, which may be more difficult to perform in rabbits [1]. Total intravenous anesthesia (TIVA) can be used as an alternative to inhalant anesthesia by continuous administration of intravenous anesthetic agents. Ketamine is a dissociative agent, which is usually administered intramuscularly or intravenously for sedation or induction of anesthesia in rabbits following sedative/ analgesic premedications. In order to improve muscle relaxation, phenothiazines (acepromazine), α_2_-agonists (xylazine, medetomidine, dexmedetomidine), benzodiazepines (diazepam, midazolam) are usually administered in combination with ketamine [2]. Sympathetic nervous system-stimulating effects of ketamine typically results in an increase in heart rate and arterial blood pressure [3]. Although ketamine, even at subanesthetic doses, has analgesic properties but does not produce muscle relaxation and prolonged recovery from anesthesia may occur if high doses are administered [2].

Propofol is a phenol derivative unrelated to barbiturates, that induces rapid and smooth anesthesia of short duration. Propofol has no analgesic action and causes dose-dependent hypotension and respiratory depression. Intense hypotension and hypoxemia following the prolonged administration of propofol alone in rabbits have been reported [4].

Ketofol, a ketamine–propofol admixture has been used for induction or maintenance of anesthesia in humans [5, 6], dogs [7–10], cats [11], horses [12] and rabbits [13–15]. Propofol and S(+)-ketamine combination has been evaluated for TIVA in acepromazine-buprenorphine premedicated rabbits [16].

Human studies have suggested that ketamine and propofol may induce opposite effects on cardiopulmonary functions [17, 18]. Total intravenous anesthesia using ketamine-propofol combination, may hypothetically minimize hypotension and respiratory depression, while enhancing the analgesic effect during intraoperative and the immediate recovery period. A recent experimental study in rabbits indicated that premedication with medetomidine, midazolam or morphine, can significantly decrease the maintenance dose of ketofol infusion [15].

Intravenous CRIs of several anesthetic, sedative and analgesic drugs have been evaluated for balanced anesthetic protocols and TIVA in rabbits [19–24]. Drugs used as CRIs for balanced anesthesia can be titrated to effect, which could result in smaller doses of each individual drug to be used (being used). In order to reach an effective plasma concentration more rapidly, an intravenous loading dose is commonly administered immediately before the start of the CRI [25].

To the authors’ knowledge, the effects of constant rate infusion of sedative/ analgesic drugs on ketofol anesthesia have not been reported previously in rabbits. In the present study, rabbits received a constant rate infusion of midazolam, fentanyl or dexmedetomidine to compare their effects on the maintenance dose of ketofol anesthesia. We hypothesized that continuous infusion of these drugs would decrease the maintenance doses of ketofol in rabbits. The primary objective of this study was to determine the appropriate doses of ketamine-propofol combination required for induction and maintenance of anesthesia during CRIs of midazolam, fentanyl or dexmedetomidine in rabbits. The secondary objective was to compare the cardiopulmonary effects of these drug combinations at similar anesthetic depth.

## Materials and methods

After obtaining approval from the University Animal Care and Use Committee (Protocol number 9,560,918/2020), 8 female New Zealand White rabbits (*Oryctolagus Cuniculus*), aged 5–7 months and weight 2.3 ± 0.2 kg (range 2.0–2.7 kg) were enrolled in the present study. Rabbits were purchased from laboratory animal center of Shiraz University of Medical Science and determined to be healthy as judged by physical examination (including heart rate, respiratory rate, rectal temperature, thoracic auscultation, mucous membrane color, pulse quality and the presence or absence of an ocular or nasal discharge).

Rabbits were housed in a controlled environment with a temperature of 20–22 °C and a 12 h day/night cycle. They were kept in pairs in stainless steel cages and had free access to standard rabbit pellets, alfalfa and tap water until 1 h before the experiment and underwent a period of acclimation of one week before the study. All experiments were conducted between 8 and 12 am. Each rabbit received a constant rate infusion of either midazolam (treatment KPM), fentanyl (treatment KPF), dexmedetomidine (treatment KPD), or saline (treatment KPS) in a randomized fashion (http://www.randomization.com, Accessed 10th November 2020). Each rabbit was administered all treatments with an interval of at least 7 days between treatments.

On the day of each experiment, the rabbits were restrained by lightly wrapping with a towel which allowed for more comfortable positioning while still preventing gross movements. Thirty minutes before auricular artery and vein catheterization, the eutectic mixture of lidocaine and prilocaine (Xyla-P® Cream, Tehran Chemie Pharmaceutical CO, Tehran, Iran) was applied on the skin of the both ears of each rabbit.

Before induction, the marginal ear vein on one side and central auricular artery on the opposite side were catheterized using 24 G over-the-needle catheters (Angiocatheter, Becton-Dickinson, USA) which were used for IV drug infusion and fluid administration, and collection of blood samples for blood gas analysis, respectively. The ketofol (as a 1:1 mixture) was prepared immediately before use, by diluting 2 mL of ketamine (Rotexmedica Germany, 50 mg/mL) in 8 mL normal saline, then adding 10 mL of propofol (Fresenius Kabi, Germany, 10 mg/mL) in a 20-mL syringe to acquire a solution of 5 mg/mL ketamine and 5 mg/mL propofol.

Animals were preoxygenated with 100% oxygen through a face mask for 3–5 min prior to and during the induction of anesthesia. Anesthesia was induced with slow intravenous (IV) injection of ketofol at a rate of approximately 10 mg/kg/min until an adequate plane of anesthesia for successful endotracheal intubation (loss of jaw tone, absence of resistance to the protraction of the tongue and absence of swallowing and gag reflexes) was achieved. Following laryngeal desensitization with 0.2 mL lidocaine (Caspian Pharmaceutical Co, Rasht, Iran, 20 mg/mL), trachea was intubated with an uncuffed tube (3 mm I.D.) by the same person using a videoendoscopic system.

If post-induction apnea (defined as an absence of spontaneous breathing for longer than 30 s) occurred, the lungs were ventilated at a rate of 4 breaths/min using the reservoir bag, until spontaneous breathing resumed. If the depth of anesthesia was not adequate for endotracheal intubation (the presence of jaw tone and/ or laryngeal reflex), small incremental doses of ketofol (2 mg/kg, IV) were administered as necessary. The total ketofol dose (total mg dose for both ketamine and propofol) required for intubation (i.e., the anesthetic induction dose) was recorded.

Following endotracheal intubation, the endotracheal tube was attached to a small animal anesthesia machine (Fabius, Drager Medical, AG & Co. KGaA, Germany) with a non-rebreathing system (Mapleson F) and the oxygen flow set at 1 L/min. Anesthetized rabbits were positioned in right lateral recumbency on a warm water pad (approximately 40 ºC) and allowed to breathe spontaneously.

Immediately following tracheal intubation, an IV infusion of ketofol (0.6 mg/kg/min [0.3 mg/kg/min of each drug]) and treatment drugs (midazolam, fentanyl, dexmedetomidine or saline) were started simultaneously using two calibrated syringe pump (JMS, Syringe pump, Japan). Treatments were as follows: treatment KPM (midazolam [5 mg/mL, Exir, Boroujerd, Iran]: loading dose 0.3 mg/kg; CRI 0.3 mg/kg/hr), treatment KPF (fentanyl [Feniject® 0.5, 50 µ/mL, Aburaihan Pharmaceutical Co., Tehran, Iran]: loading dose 6 µg/kg; CRI 6 µg/kg/hr), treatment KPD (dexmedetomidine [Medonex® 200, 100 mcg/mL, Exir Pharmaceutical Co, Boroojerd, Iran]: loading dose 3 µg/kg; CRI 3 µg/kg/hr), or treatment KPS (saline: loading dose & CRI)(Table 1) [33].

**Table 1:** Treatment groups in rabbits anesthetized with ketofol (combination of ketamine and propofol) and received either midazolam [treatment KPM], fentanyl [treatment KPF], dexmedetomidine [treatment KPD] or saline [treatment KPS] constant rate infusions (CRIs)

Treatments	Induction	Maintenance*	CRIs
**KPM**	ketofol	ketofol infusion	Midazolam LD: 0.3 mg/kg; CRI: 0.3 mg/kg/hr
**KPF**	ketofol	ketofol infusion	Fentanyl LD: 6 µg/kg; CRI: 6 µg/kg/hr; CRI
**KPD**	ketofol	ketofol infusion	Dexmedetomidine LD: 3 µg/kg; CRI: 3 µg/kg/hr; CRI
**KPS**	ketofol	ketofol infusion	Saline

*Starting infusion rate 0.3 mg/kg/min of each drug

LD: Loading dose

The loading doses of midazolam, fentanyl or dexmedetomidine were diluted in sterile 0.9% saline to a final volume of 0.5 mL and administered slowly over one minute. For continuous infusion, all treatment drugs were diluted with sterile 0.9% saline and administered at a rate of 5 mL/kg/hr. Rabbits in the KPS treatment were administered the same volume of saline for loading dose and CRI. Continuous infusion of ketofol and the treatment drugs were administered for 70 and 60 min, respectively. Initial dose of ketofol (0.3 mg/kg/min of each drug) was continued for 15 min before the first stimulus was applied. Then, the pedal withdrawal reflex was evaluated by a toe pinch in the pelvic limb with a hemostat clamped to the third ratchet. The same observer, who was unaware of the treatment administered to each rabbit, evaluated the pedal withdrawal reflex at 10-minute intervals for 70 min after initiation of drug infusion [10, 15]. The stimulus was applied for 5 s or until a positive response (withdrawal of the stimulated limb) was observed. If no response to stimulation occurred, ketofol infusion rate was decreased by 0.1 mg/kg/min (0.05 mg/kg/min of each drug), and if there was a positive response, the infusion rate was increased by 0.1 mg/kg/min [26]. Ketofol infusion rate was held constant for a 10-minute equilibration time before the next stimulation was applied. If spontaneous movement or swallowing was observed, ketofol was given as a bolus of 2 mg/kg intravenously. The KP infusion was discontinued after 70 min. The total dose of intravenous ketofol (mg/kg/min) used for maintenance of anesthesia (total mg dose for both drugs) was calculated for each rabbit by adding bolus doses to total drug dose administered as infusion.

The following physiologic variables were recorded at 5-minute intervals throughout anesthesia: heart rate (HR), respiratory rate (*f*_R_), oxygen saturation of hemoglobin (SpO_2_) using pulse oximetry (placing the probe on the hindlimb toe-web), end-tidal carbon dioxide tension (PE′CO_2_-using main-stream capnography), non-invasive systolic arterial blood pressure (SAP) and rectal temperature (RT) (PM 9000, Mindray, China). Indirect systolic arterial blood pressure was monitored with a Doppler ultrasound technique (ultrasonic Doppler flow detector -Model 811-B; Parks Medical Electronics, OR, USA), a cuff with a width approximately 40% of the circumference of the limb placed on the carpus (approximately level with the thoracic inlet) and sphygmomanometer. Arterial blood samples were collected from auricular artery into a pre-heparinized 1 mL syringe at 10, 35 and 70 min after induction and analyzed immediately to determine packed cell volume, hemoglobin concentration, partial pressures of oxygen (PaO_2_) and carbon dioxide (PaCO_2_), pH, bicarbonate (HCO_3_^−^) and base excess (BE) using a blood gas analyzer (OPTI Medical System Inc., USA).

All physiologic variables were recorded before evaluation of response to noxious stimulation (pedal withdrawal reflex). Five minutes after discontinuation of ketofol infusion (time 75 min from start of drug infusion), a total of 4 rabbits in each treatment were administered a specific antagonist intravenously (i.e., KPM: flumazenil [Anexate®, 0.5 mg/5 mL, Cenexi SAS, Fontenay-sous-Bois, France] 0.15 mg/kg; KPF: naloxone [0.4 mg/mL, Caspian Tamin Pharmaceutical Co, Rasht, Iran] 0.04 mg/kg; KPD: atipamezole [Antisedan®, 5 mg/mL, Orion Pharma, Finland] 15 µg/kg) and the rabbits were allowed to recover from anesthesia. Rabbits in KPS treatment received the same volume of saline. When the swallowing reflex returned, the tracheal tube was removed and the rabbits were observed continuously throughout the recovery period. The time intervals extubation, first head lift and sternal recumbency were recorded. Time to extubation was defined as the time from the end of ketofol infusion to extubation, time to first head lift was defined as the time from the end of ketofol infusion to when the rabbit could lift its head and time to sternal recumbency was defined as the time from the end of ketofol infusion to when the rabbit assumed sternal recumbency. After completion of this study, the rabbits were adopted as pet by interested students.

### Statistical analysis

Based on data from a previous study [14], the required sample size was six animals to achieve an alpha value of 5% and power of 80% for detecting a large effect on maintenance doses of ketofol (G*Power 3.1.9.7; University of Düsseldorf, Germany). Normality of the data was assessed using the Shapiro-Wilk test. Physiologic data (HR, SAP, *f*_R_, PE′CO_2_, RT, SpO_2_, PaO_2_, PaCO_2_, BE, bicarbonate concentration and pH) were analyzed using an ANOVA for repeated-measures, with time and treatment as factors. When significant treatment effects were noted, Tukey’s test was used for post hoc comparisons. Ketofol dose (induction and maintenance) and recovery times were analyzed with one-way analysis of variance (ANOVA) with Tukey’s test for post hoc analysis. Statistical analysis was performed with standard computer software (SPSS version 24 for Windows, IBM Corp, Armonk, NY.). Data are presented as mean ± standard deviation. Statistical significance was identified at *p* < 0.05.

## Results

Induction was rapid and excitement-free with a smooth transition to unconsciousness and all rabbits were successfully intubated following induction. Ketofol induction dose did not differ significantly among the four treatments. The total dose of ketofol used to maintain anesthesia in treatments KPM and KPD (1.0 ± 0.1 mg/kg) were significantly lower as compared to KPS treatment (1.3 ± 0.1 mg/kg) (*p* < 0.05). Ketofol maintenance dose was no significantly different in KPF compared to KPM, KPD and KPS treatments (Table 2). The ketofol infusion rate decreased in KPM, KPF and KPD treatments by 23%, 15% and 23%, respectively. The amount of supplemental boluses of ketofol required to prevent voluntary movement or spontaneous swallowing during ketofol infusion was significantly higher in KPS (24 boluses [13.2 ± 8.4 mg/kg]) as compared to KPM (5 boluses [1.4 ± 1.6 mg/kg]), KPF (19 boluses [4.4 ± 4.1 mg/kg]) and KPD (6 boluses [2.1 ± 3.6 mg/kg]) treatments (*p* = 0.001). Administration of supplemental boluses of ketofol were required in 4 rabbits in KPM, 6 rabbits in KPF, 3 rabbits in KPD and all rabbits in KPS treatments. The number of rabbits required supplemental boluses of ketofol were significantly higher in KPS compared to the other treatments (Fisher’s exact test, *p* = 0.046).

**Table 2:** Anesthetic induction and maintenance doses of an intravenous combination of ketamine (5 mg/mL) and propofol (5 mg/mL) in eight rabbits received either midazolam [treatment KPM], fentanyl [treatment KPF], dexmedetomidine [treatment KPD] or saline [treatment KPS] constant rate infusions (CRIs) for 70 min. Dose results are presented as total mg dose for both drugs. Data are presented as mean ± standard deviation

Treatments	KPM	KPF	KPD	KPS
**Ketofol induction dose** **(mg/kg)**	14.9 ± 1.8	15.0 ± 1.9	15.5 ± 2.4	14.7 ± 3.4
**Ketofol maintenance dose (mg/kg/min)**	1.0 ± 0.1*	1.1 ± 0.3	1.0 ± 0.1*	1.3 ± 0.1

* Significantly different from saline treatment (*p* < 0.05)

Generally, heart rate (beats/min) increased following ketofol induction, which was significant only in KPM and KPD treatments (*p* < 0.05). Ten minutes after induction, HR in KPD treatment was significantly lower compared to KPS treatment (Table 3). When the data were averaged to calculate the overall mean HR during 70 min ketofol anesthesia, the lowest HR was observed with treatment KPD (197 ± 32 beats/min) followed by treatment KPF (209 ± 38 beats/min), and both were significantly lower than treatment KPS (222 ± 38 beats/min) (*p* < 0.05). The overall mean HR was significantly higher in treatment KPM (215 ± 32 beats/min) when compared with treatment KPD (197 ± 32 beats/min). There were no differences among the treatments in SAP (*p* > 0.05). However, the overall SAP were lower in treatments KPF (105 ± 32 mmHg) and KPD (104 ± 18 mmHg) when compared with treatments KPM (125 ± 15 mmHg) and KPS (117 ± 39 mmHg).

**Table 3:** Heart rate (HR) and respiratory rate (fR), systolic arterial pressure (SAP) and rectal temperature (RT) in rabbits (*n* = 8) anaesthetized with an intravenous combination of ketamine (5 mg mL^− 1^) and propofol (5 mg mL^− 1^) and received either midazolam [treatment KPM], fentanyl [treatment KPF], dexmedetomidine [treatment KPD] or saline [treatment KPS] infusion for 70 min. Time points − 10, before induction of anesthesia; 0, immediately after induction of anesthesia. Data are presented as mean ± standard deviation

Variable / Treatment		Time (minutes) after starting ketofol infusion and CRIs															
		–10	0	5	10	15	20	25	30	35	40	45	50	55	60	65	70
**HR (beats min** ^–1^ **)**																	
KPM	185 ± 32	252 ± 9*	240 ± 18*	227 ± 27	217 ± 38	229 ± 27	218 ± 27	215 ± 24	206 ± 25	212 ± 26	207 ± 30	204 ± 34	199 ± 29	213 ± 37	207 ± 36	203 ± 35	
KPF	196 ± 30	245 ± 20	231 ± 31	223 ± 28	206 ± 35	208 ± 32	215 ± 33	219 ± 36	186 ± 23	216 ± 42	199 ± 50	200 ± 51	195 ± 47	205 ± 44	196 ± 38	199 ± 36	
KPD	179 ± 28	252 ± 11*	232 ± 31	207 ± 33^‡^	201 ± 38	205 ± 37	195 ± 38	197 ± 37	191 ± 37	194 ± 34	187 ± 37	188 ± 36	182 ± 39	186 ± 41	178 ± 47	184 ± 40	
KPS	196 ± 24	250 ± 13	252 ± 3	250 ± 12	241 ± 18	240 ± 18	232 ± 33	226 ± 38	211 ± 52	214 ± 40	217 ± 46	209 ± 40	210 ± 48	205 ± 45	204 ± 48	199 ± 43	
**fR (breaths min** ^–1^ **)**																	
KPM	141 ± 51	23 ± 10*	29 ± 11*	31 ± 17✝*	30 ± 15✝*	27 ± 13*	26 ± 13*	24 ± 11*	24 ± 13*	21 ± 11*	19 ± 10*	18 ± 9*	17 ± 8*	19 ± 9*	21 ± 7*	18 ± 8*	
KPF	149 ± 38	28 ± 16*	16 ± 14*	13 ± 7*	14 ± 7*	13 ± 6*	15 ± 10*	14 ± 6*	15 ± 9*	16 ± 9*	19 ± 13*	12 ± 5*	13 ± 6*	14 ± 5*	14 ± 5*	19 ± 5*	
KPD	175 ± 42	32 ± 14*	35 ± 8✝*	32 ± 9✝*	28 ± 10✝*	30 ± 14✝*	24 ± 11*	25 ± 10*	23 ± 9*	21 ± 9*	20 ± 8*	20 ± 8*	19 ± 10*	22 ± 12*	17 ± 9*	19 ± 8*	
KPS	149 ± 37	30 ± 7*	36 ± 11✝*	28 ± 12*	25 ± 8*	21 ± 7*	21 ± 9*	23 ± 15*	20 ± 7*	21 ± 10*	22 ± 9*	20 ± 8*	21 ± 6*	19 ± 6*	18 ± 7*	17 ± 6*	
**SAP (mmHg)**																	
KPM		138 ± 20	131 ± 23	127 ± 16	121 ± 8	118 ± 15	119 ± 13	119 ± 13	127 ± 13	123 ± 11	116 ± 12	124 ± 16	125 ± 11	129 ± 17✝	125 ± 14	132 ± 18	
KPF		113 ± 35	105 ± 39	110 ± 43	112 ± 36	111 ± 35	112 ± 40	117 ± 40	110 ± 34	107 ± 31	102 ± 32	98 ± 28	105 ± 22	86 ± 15	93 ± 22	98 ± 27	
KPD		108 ± 12	109 ± 9	104 ± 21	102 ± 14	97 ± 19	108 ± 18	101 ± 23	102 ± 23	105 ± 15	104 ± 25	104 ± 21	97 ± 16	102 ± 18	109 ± 24	106 ± 20	
KPS		130 ± 37	127 ± 34	117 ± 30	121 ± 36	116 ± 38	119 ± 49	122 ± 52	121 ± 49	115 ± 45	123 ± 42	112 ± 38	108 ± 37	114 ± 45	103 ± 33	104 ± 32	
**RT (°C)**																	
KPM	39.2 ± 0.4	38.7 ± 0.6	38.7 ± 0.5	38.6 ± 0.6	38.5 ± 0.6	38.4 ± 0.6	38.3 ± 0.6	38.2 ± 0.6*	38.1 ± 0.6*	38.0 ± 0.6*	37.9 ± 0.6*	37.9 ± 0.5*	37.8 ± 0.5*	37.7 ± 0.6*	37.6 ± 0.6*	37.6 ± 0.5*	
KPF	39.1 ± 0.3	38.7 ± 0.4	38.4 ± 0.4*	38.3 ± 0.4*	38.2 ± 0.4*	38.1 ± 0.3*	38.0 ± 0.4*	37.9 ± 0.3*	37.8 ± 0.3*	37.7 ± 0.4*	37.6 ± 0.4*	37.5 ± 0.4*	37.4 ± 0.4*	37.3 ± 0.4*	37.3 ± 0.4*	37.2 ± 0.5*	
KPD	38.9 ± 0.4	38.5 ± 0.5	38.4 ± 0.5	38.3 ± 0.5	38.1 ± 0.6	38.0 ± 0.7	37.9 ± 0.7	37.8 ± 0.7	37.7 ± 0.7*	37.6 ± 0.7*	37.6 ± 0.7*	37.5 ± 0.7*	37.4 ± 0.7*	37.3 ± 0.7*	37.3 ± 0.7*	37.2 ± 0.7*	
KPS	38.9 ± 0.3	38.4 ± 0.4	38.4 ± 0.4	38.2 ± 0.5	38.1 ± 0.4	38.0 ± 0.4	37.9 ± 0.5	37.8 ± 0.5*	37.7 ± 0.6*	37.6 ± 0.6*	37.5 ± 0.6*	37.4 ± 0.7*	37.4 ± 0.7*	37.3 ± 0.0.7*	37.2 ± 0.7*	37.1 ± 0.8*	

* Significantly different from **− 1**0 minutes within the same treatment (*p* < 0.05)

✝ Significantly different from fentanyl treatment (*p* < 0.05)

‡ Significantly different from saline treatment (*p* < 0.05)

Respiratory rate (breaths/min) was significantly decreased in all treatments following induction of anesthesia with ketofol (*p* < 0.05) and remained lower than time 0 (baseline) throughout ketofol anesthesia. Respiratory rate was significantly lower in treatment KPF as compared to treatment KPM (at 10 to 15 min), treatment KPD (at 5 to 20 min) and treatment KPS (at 5 min) (Table 3). In addition, apnea during ketofol infusion was observed in 0, 2, 4 and 7 rabbits in KPD, KPM, KPS and KPF treatments, respectively, with a more prolonged period of apnea in KPF treatment. The occurrence of apnea was significantly higher in KPF compared to the other treatments (Fisher’s exact test, *p* = 0.002).

There were no significant difference over time among treatments in SpO_2_, PaO_2_, pH, HCO_3_^−^ and BE. PE′CO_2_ significantly increased in treatments KPM (at 65 min), in KPF (at 35 to 60 min), in KPD (at 70 min) and in KPS (at 60 to 70 min) compared with baseline values (Table 4). PE′CO_2_ was significantly higher in KPF from 5 to 55 min when compared to other treatments (*p* < 0.05). The overall PE′CO_2_ was higher in treatments KPF (48 ± 14) compared with KPM (34 ± 10 mmHg), KPD (29 ± 8 mmHg) and KPS (31 ± 9 mmHg) during the 70 min period (*p* = 0.000). PE′CO_2_ was also significantly higher in treatment KPM than in KPD and KPS treatments. In treatment KPF, arterial pH was significantly lower (7.16 ± 0.06) compared to KPM (7.28 ± 0.06), KPD (7.29 ± 0.08) and KPS (7.33 ± 0.04) after 10 min of anesthesia and compared to KPS after 35 min of anesthesia (7.15 ± 0.14 vs. 7.35 ± 0.10). PaCO_2_ was significantly higher in KPF treatment compared to KPS after 10 and 35 min of anesthesia.

**Table 4:** Hemoglobin oxygen saturation (SpO_2_), end-tidal carbon dioxide (P_E′_CO_2_), pH, arterial partial pressure of oxygen (PaO_2_), arterial partial pressure of carbon dioxide (PaCO_2_), bicarbonate concentration (HCO_3_^−^) and base excess (BE) in rabbits (*n* = 8) anaesthetized with an intravenous combination of ketamine (5 mg mL^− 1^) and propofol (5 mg mL^− 1^) and received either midazolam [treatment KPM], fentanyl [treatment KPF], dexmedetomidine [treatment KPD] or saline [treatment KPS] infusion for 70 min. Time point 0, immediately after induction of anesthesia. Data are presented as mean ± standard deviation

Variable / Treatment		Time (minutes) after starting ketofol infusion and CRIs															
		0	5	10	15	20	25	30	35	40	45	50	55	60	65	70	
**SpO**_**2**_ **(%)**																	
KPM	99 ± 1	98 ± 2	99 ± 1	99 ± 1	99 ± 1	99 ± 1	99 ± 1	99 ± 1	99 ± 1	99 ± 1	99 ± 1	99 ± 1	99 ± 1	99 ± 1	98 ± 2		
KPF	96 ± 4	96 ± 4	94 ± 6	97 ± 3	98 ± 1	96 ± 4	98 ± 2	98 ± 2	98 ± 2	98 ± 1	96 ± 3	97 ± 3	98 ± 2	98 ± 2	98 ± 1		
KPD	98 ± 2	98 ± 2	99 ± 1	99 ± 1	99 ± 1	99 ± 1	99 ± 1	99 ± 1	99 ± 1	99 ± 1	99 ± 1	99 ± 1	99 ± 1	99 ± 1	99 ± 1		
KPS	98 ± 1	96 ± 4	96 ± 4	97 ± 3	98 ± 1	99 ± 1	99 ± 1	98 ± 2	99 ± 1	99 ± 1	98 ± 2	98 ± 2	99 ± 1	99 ± 1	99 ± 1		
**P**_**E′**_ **CO**_**2**_ **(mmHg)**																	
KPM	28 ± 10	29 ± 10	27 ± 3	29 ± 5	30 ± 5	33 ± 4	33 ± 5	37 ± 9	36 ± 12	35 ± 2	36 ± 4	35 ± 9	41 ± 9	44 ± 16*	44 ± 15		
KPF	29 ± 8	41 ± 8‡	42 ± 8‡	46 ± 12‡	45 ± 12‡	45 ± 8‡	49 ± 10‡	55 ± 16‡*	54 ± 13‡*	60 ± 22‡*	53 ± 14‡*	52 ± 9‡*	54 ± 12*	51 ± 12	47 ± 10		
KPD	24 ± 7	22 ± 5	22 ± 7	24 ± 4	26 ± 4	28 ± 5	29 ± 5	31 ± 4	32 ± 7	33 ± 8	29 ± 7	34 ± 10	34 ± 10	36 ± 9	37 ± 9*		
KPS	21 ± 3	25 ± 6	27 ± 5	27 ± 7	28 ± 6	29 ± 5	28 ± 9	32 ± 6	33 ± 8	35 ± 10	34 ± 8	33 ± 7	37 ± 13*	36 ± 11*	37 ± 11*		
**pH**																	
KPM			7.28 ± 0.06					7.29 ± 0.09							7.24 ± 0.06		
KPF			7.16 ± 0.06 ‡					7.15 ± 0.14 §							7.31 ± 0.08		
KPD			7.29 ± 0.08					7.30 ± 0.09							7.30 ± 0.03		
KPS			7.33 ± 0.04					7.35 ± 0.10							7.27 ± 0.08		
**PaO**_**2**_ **(mmHg)**																	
KPM			260 ± 68					214 ± 80							239 ± 74		
KPF			133 ± 70					227 ± 71							232 ± 102		
KPD			211 ± 85					229 ± 67							181 ± 62		
KPS			202 ± 67					218 ± 84							218 ± 83		
**PaCO**_**2**_ **(mmHg)**																	
KPM			48.3 ± 7.0					53.8 ± 18.9							58.1 ± 12.6		
KPF			57.7 ± 15.2§					69.9 ± 16.0§							53.6 ± 13.8		
KPD			43.8 ± 5.1					50.7 ± 17.1							53.3 ± 4.7		
KPS			40.6 ± 3.1					42.2 ± 14.0							59.8 ± 20.7		
**HCO**_**3**_ **(mmol/L)**																	
KPM			21.5 ± 1.6					23.9 ± 3.3							24.1 ± 4.6		
KPF			19.6 ± 4.2					25.4 ± 6.3							26.2 ± 4.4		
KPD			20.5 ± 4.1					23.6 ± 3.7							25.9 ± 3.5		
KPS			21.0 ± 2.1					22.1 ± 3.3							25.9 ± 4.6		
**BE (mmol/L)**																	
KPM			–4.8 ± 1.5					–2.8 ± 1.5							–3.6 ± 4.2		
KPF			–8.7 ± 3.8					–4.2 ± 4.6							–0.5 ± 3.9		
KPD			–5.6 ± 4.8					–2.9 ± 3.0							–1.1 ± 3.1		
KPS			–4.1 ± 2.5					–3.0 ± 3.0							–1.6 ± 2.9		

*Significantly different from induction time (Time 0) within the same treatment (*p* < 0.05)

‡Significantly different from other treatments (*p* < 0.05)

§Significantly different from saline treatment (*p* < 0.05)

All treatments had similar changes in rectal temperature which were decreased significantly over time in KPM (from T30 to T70), KPF (from T5 to T70), KPD (from T35 to T70) and KPS (from T30 to T70) treatments compared to baseline values (Time − 10) (*p*<0.05, Table 3). After discontinuation of ketofol infusion, there were no statistically significant differences among the treatments with respect to intervals to extubation, head lift and sternal recumbency (*p* > 0.05; Table 5). Recovery times did not change following specific antagonist administration in any treatments. Recovery from anesthesia, with or without administration of antagonist, was considered smooth, calm and uneventful in all treatments and no complications were encountered during the 4-weeks observation period in the present study.

**Table 5:** Recovery times in rabbits (*n* = 8) anaesthetized with an intravenous combination of ketamine (5 mg/mL) and propofol (5 mg/mL) and received either midazolam [treatment KPM], fentanyl [treatment KPF], dexmedetomidine [treatment KPD] or saline [treatment KPS] infusion for 70 min. Variables were timed from the end of ketofol infusion. Data are presented as mean ± standard deviation

Variable	KPM	KPF	KPD	KPS
Time to extubation (minutes)	10.3 ± 5.6	6.6 ± 2.4	8.6 ± 4.3	10.0 ± 3.5
Time to head lift (minutes)	23.5 ± 9.9	17.3 ± 6.8	20.1 ± 9.7	25.2 ± 6.2
Time to sternal recumbency (minutes)	50.3 ± 15.3	39.8 ± 17.3	46.5 ± 18.1	49.0 ± 6.7

## Discussion

Administration of midazolam and dexmedetomidine CRIs significantly decreased the ketofol infusion rate without significantly prolonging recovery times. Maintenance dose for ketofol was not significantly reduced in treatment KPF compared with treatment KPS. A higher infusion rate of fentanyl may be required to provide ketofol-sparing effects; however, this may increase the risk of opioid-induced ileus, a serious post-operative complication, in rabbits [27]. In a recent study, although premedication with morphine (another full µ-opioid agonist) decreased the ketofol infusion rate during maintenance of anesthesia, induction dose of ketofol was not affected by morphine administration [15]. In the present study, ketofol infusion rate decreased in KPM, KPF and KPD treatments by 23%, 15% and 23%, respectively. Terada et al. (2014) reported that dexmedetomidine CRI (3.5 µg/kg/hr) reduced propofol requirement by 11% in rabbits [28].

In the present study, ketofol infusion provided more stable depth of anesthesia when combined with a CRI of midazolam, fentanyl or dexmedetomidine compared with rabbits administered saline, as indicated by the lower amount of ketofol required as bolus doses to prevent spontaneous movement. Premedication with medetomidine, midazolam or morphine produces similar effects in rabbits [15].

In a recent study, a similar ketofol infusion rate was used to maintain anesthesia in rabbits premedicated with intramuscular saline but infusion rates in rabbits premedicated with medetomidine, midazolam or morphine were in the range of 0.6 ± 0.2 mg/kg/min [15]. However, in that study, a higher dose of drugs were used as intramuscular premedication (midazolam 1 mg/kg, morphine 1 mg/kg and medetomidine 100 µg/kg) compared to the present study (loading dose plus CRI: midazolam 0.6 mg/kg, fentanyl 12 µg/kg and dexmedetomidine 6 µg/kg), which may have contributed to the relatively lower maintenance dose of ketofol needed in that study.

The overall mean HR were significantly lower in treatments KPF and KPD compared to treatment KPS. A lower mean HR in KPF compared to KPS could be attributed to infusion of fentanyl, a short-acting full µ opioid agonist, that causes bradycardia through medullary vagal stimulation [29, 30]. A significant decrease in heart rate was observed following repeated boluses of fentanyl (5 mg/kg IV) in healthy rabbits anesthetized with propofol [31]. Vagally-induced bradycardias can be reversed by the administration of anticholinergics such as atropine and glycopyrrolate without affecting the analgesia, especially when bradycardia is associated with hypotension [32]. Atropine may not be as effective as glycopyrrolate because of its rapid metabolism via a plasma esterase, atropinase, in rabbits [33]. A significant reduction in HR has been reported following intramuscular administration of dexmedetomidine-ketamine compared to midazolam-ketamine in rabbits [34]. Dexmedetomidine lowers the HR due to a baroreceptor (vagally)–mediated reflex and decreased sympathetic tone [35]. The ketamine component of ketofol may have counteracted some of the bradycardic effects produced by fentanyl and dexmedetomidine through its sympathomimetic activity [3].

Lower mean SAP was observed in KPF and KPD treatments as compared to KPM and KPS treatments. Fentanyl-induced hypotension is generally due to reduction in HR rather than peripheral vasodilation and pre-medication with anticholinergics could be expected to prevent hypotension [36]. Biphasic blood pressure changes (initial hypertension followed by prolonged hypotension) usually occurs after α_2_-agonists administration [37]. Hypotensive phase is due to decreased vascular resistance with continued bradycardia.

Marked reduction in *f*_R_ following anesthetic induction can be explained by the respiratory depressant effect of ketofol at the medullary respiratory center [16], which is thought to be dose-dependent [13]. Although the respiratory rate was decreased in all treatments, hypoxemia was prevented by high-inspired oxygen provided during anesthesia. The higher P_E′_CO_2_ encountered during ketofol anesthesia in KPF treatment can be attributed to the profound respiratory depressant effects of opioids [20, 30]. Fentanyl has an additive respiratory depressant effect when used in combination with other anesthetics that may result in severe hypercarbia or even apnea [36]. More rabbits in KPF treatment occasionally required manual ventilation during anesthetic maintenance due to apnea. On average, rabbits in KPD treatment tended to have a higher *f*_R_ and a lower P_E′_CO_2_ when compared to other treatments. The respiratory depressant effect of α_2_-agonists is considered to be minimal in healthy animals [38]. Lower pH values that observed in treatment KPF was most likely of respiratory origin, because this change was accompanied by increased P_E′_CO_2_ (hypercarbia).

In the present study, despite application of an external heat source to minimize temperature loss during anesthesia, a small but significant reduction in rectal temperature was observed in all treatments, with a longer duration in rabbits given KPF treatment. Perianesthetic hypothermia as a common complication in small animal patients occurs mainly due to heat loss in excess to metabolic heat production or resetting of thermoregulatory mechanisms. When severe, hypothermia may lead to a significant reduction of anesthetic requirement, prolonged recovery, surgical wound infection and cardiac arrhythmias [39]. Therefore, use of externally applied supplemental heat source is recommended in the perioperative period.

Since administration of specific antagonists did not result in shortening the recovery times, the antagonist doses used might have been insufficient to reverse agonist drugs in rabbits. Another possible explanation may be that the residual effects of ketofol infusion are the principal determinant of recovery times. Although ketamine metabolism to its active metabolite norketamine occurs primarily in the liver, extensive extrahepatic metabolism may also occur in rabbit’s lungs [40]. Norketamine is metabolized more slowly than the parent drug and can potentially accumulate and influence recovery time after prolonged infusions. It is also possible that the active metabolites of some drugs used as CRI may be responsible for prolonged recovery times in the present study. In a recent study, a prolonged sedation unresponsive to flumazenil administration has been reported following midazolam infusion in sevoflurane-anesthetized cats, which could be attributed to the accumulation of active metabolite of midazolam (1-hydroxymidazolam), that peaked after stopping the infusion [41].

Flumazenil, naloxone and atipamezole are considered competitive antagonists and timing of their administration may be another factor influencing the recovery times. In other words, the dose of antagonist drugs may need to be adjusted according to the time delay following administration of agonist drugs. In the present study, antagonists were administered at 15 min after termination of agonist CRI, which corresponds to 5 min after termination of ketofol infusion. However, different timing of atipamezole administration (20 vs. 40 min) in cats anesthetized with intramuscular ketamine-buprenorphine-midazolam-medetomidine did not result in significant differences in recovery times [42]. Further studies are required to establish the optimum dosage and timing of administration of agonists (flumazenil, naloxone and atipamezole) in rabbits undergoing ketofol anesthesia supplemented with CRIs of benzodiazepines, opioids or α_2_-agonists CRIs.

Although it is generally recommended to administer atipamezole intramuscularly to prevent arterial hypotension [36], in the present study intravenous route was chosen to bypass tissue absorption and eliminate inter-individual variation that may occur from unequal absorption of atipamezole. In addition, other agonists (i.e., flumazenil and naloxone) were also administered IV. Reversal of α_2_-agonists or opioids by selective antagonist also reverses any remaining associated analgesic effects. Therefore, in surgical cases, suitable analgesia should be provided with other drugs before antagonists are administered.

## Conclusion

In the present study, midazolam and dexmedetomidine, at the doses used, reduced ketofol infusion rates in New Zealand White rabbits and may prove to be a useful adjunct during anesthesia in this species. Fentanyl CRI did not significantly reduced ketofol infusion rates and was associated with respiratory depression and apnea. Further studies are needed to evaluate the potential cardiovascular benefits of midazolam, fentanyl or dexmedetomidine during surgery and to determine the optimal dose regimen for midazolam, fentanyl and dexmedetomidine CRIs in terms of anesthetic-sparing effects in rabbits.

## Data Availability

The datasets generated and/or analyzed during the current study are available from the corresponding author on reasonable request.

## Abbreviations

BE: Base excess
CRI: Constant rate infusion
HCO_3_^−^: Bicarbonate
LD: Loading dose
PaCO_2_: Partial pressures of carbon dioxide
PaO_2_: Partial pressures of oxygen
PE′CO_2_: End-tidal carbon dioxide tension
RT: Rectal temperature
SAP: Systolic arterial blood pressure
SpO_2_: Oxygen saturation of hemoglobin
TIVA: Total intravenous anesthesia
